# Prognostic values of m6A RNA methylation regulators in differentiated Thyroid Carcinoma

**DOI:** 10.7150/jca.41193

**Published:** 2020-07-06

**Authors:** Nizhen Xu, Jian Chen, Gaofei He, Li Gao, Deguang Zhang

**Affiliations:** Department of Head and Neck surgery, Institute of Micro-Invasive Surgery of Zhejiang University, Sir Run Run Shaw Hospital, School of Medicine, Zhejiang University, 3 East Qingchun Road, Hangzhou 310016, P.R. China.

**Keywords:** thyroid carcinoma, m6A, RNA modification, prognosis, TCGA

## Abstract

N6-methyladenosine (m6A) is the most prevalent modification of RNA in mammals. m6A RNA methylation levels are dynamically regulated by m6A RNA methylation regulators. While increasing evidence has suggested that m6A RNA methylation is vital in the initiation and progression of human carcinoma, little is known about the expression and effect of m6A RNA methylation regulators in differentiated thyroid carcinoma (DTC). Herein, we demonstrate that most of the thirteen main m6A RNA methylation regulators are differentially expressed in DTC tissues and normal thyroid tissues. Based on consensus clustering of m6A RNA methylation regulators, DTC cases were divided into two subgroups (TC1 and TC2). Compared with the TC1 subgroup, the TC2 subgroup was associated with a poorer prognosis, older age, higher T grade, higher N grade and higher TNM stage. The results indicated that alteration of m6A RNA methylation regulators was closely related to DTC. We further established a risk signature of four m6A RNA methylation regulators that could evaluate prognosis and clinicopathological features in DTC. Finally, the results of the TCGA analysis were verified by other cohorts from Gene Expression Omnibus (GEO) database. In conclusion, m6A RNA methylation regulators play a crucial part in the progression of DTC and are potentially useful for evaluating the prognosis and providing potential novel insights into treatment strategies.

## Introduction

Thyroid carcinoma is the most common malignant endocrine tumor with an incidence that has remarkably increased in the past 10 years [1‑5]. Thyroid carcinoma ranks ninth in cancer incidence worldwide and accounts for 5.1% of the total estimated cancer burden in females in 2018 6. Differentiated thyroid carcinoma (DTC), which includes papillary thyroid carcinoma (PTC) and follicular thyroid carcinoma (FTC), comprises more than 90% of all thyroid carcinomas 7, 8. Although DTC generally presents indolent behavior and has a favorable prognosis, approximately 20%-30% of DTC patients develop recurrence or distant metastasis after primary treatment. Several cases have a poor response to conventional treatment, resulting in poor prognoses 9-11.

RNA modification have widely studied in the proliferation and metastasis of human carcinoma 12, 13. The importance of messenger RNA (mRNA) plays in the post-transcriptional regulation of gene expression is well-established. In eukaryotes, N6-methyladenosine (m6A) is recognized as the most common internal chemical modification in mRNA, the process of which has been found to be dynamic and reversible 14-16. During the processes of stem cell differentiation, embryo development, neural development or stress responses, m6A can regulate the key biological process of cells through modulating mRNA stability, splicing, intracellular distribution and translation 17-20. m6A modification is dynamically regulated by methyl-transferases ('writers'), binding proteins ('readers'), and demethylases ('erasers'). The abundance, prevalence, and distribution of m6A are regulated by writers and erasers, while readers transform the m6A methylation information into a functional signal 21, 22. Writers include WT1-associated protein (WTAP), methyltransferase like 3 (METTL3), methyltransferase like 14 (METTL14), RNA binding motif protein 15 (RBM15), zinc finger CCCH-type containing 13 (ZC3H13) and KIAA1429, and readers include YTH domain-containing 1 (YTHDC1), YTH domain-containing 1 (YTHDC2), YTH N6-methyl-adenosine RNA binding protein 1 (YTHDF1), YTH N6-methyladenosine RNA binding protein 2 (YTHDF2) and heterogeneous nuclear ribonucleoprotein C (HNRNPC). Erasers include fat mass- and obesity-associated protein (FTO) and α-ketoglutarate-dependent dioxygenase alkB homolog 5 (ALKBH5) 23-25. Increasing evidence indicates that alterations of m6A regulatory genes are closely associated with obesity, infertility, immunological disease and neurological diseases. Recent studies have revealed that genetic changes and dysregulated expressions of m6A RNA methylation regulators play a crucial role in the progression of a variety of human cancers 26-28. Although previous studies have found that m6A RNA methylation regulators were related to tumor progression in different types of cancers, the expression of m6A RNA methylation regulators as well as the prognosis value in DTC has not been completely explored.

The present study analyzed the expression of 13 m6A RNA regulators in DTC with RNA sequencing data from The Cancer Genome Atlas (TCGA) and evaluated the association between m6A RNA methylation regulators and clinicopathological characteristics in DTC patients. We further established a signature with four m6A RNA methylation regulators to predict the prognosis of DTC.

## Materials and Methods

### Data source

The RNA-seq transcriptome data and clinical information of DTC patients were obtained from the Genomic Data Commons Data Portal within TCGA (https://portal.gdc.cancer.gov/) in June 2019. First, the RNA‑seq data files were merged into a matrix file using the merge script of the Perl language (http://www.perl.org/). Gene names were converted from the Ensembl ID to the matrix of the gene symbol through the Ensembl database version 84 (http://asia.ensembl.org/index.html). The downloaded data included 509 thyroid carcinoma samples and 58 normal thyroid samples. The data of 13 m6A RNA modification regulators were extracted and analyzed. The R (version 3.6.0) package 29 edgeR version 3.27.6 (r-project.org/) was used to identify genes that were differentially expressed between tumor and normal thyroid samples, with a false discovery rate <0.05 and |log_2_ fold change| >2 set as the threshold. The gene expression level based on microarray data was calculated using R package limma (version 3.40.2; bioconductor.org/packages/release/bioc/html/limma.html) with robust multiarray average (RMA) correction.

### Information of 13 m6A RNA methylation regulators and clinicopathological features

We excluded patients with incomplete clinicopathological parameters or those with missing prognostic follow-up data. A total of 425 DTC patients were enrolled in our study (Table 1). Information of 13 m6A RNA methylation regulators in DTC and normal thyroid were obtained from downloaded data, including 425 tumors and 57 normal samples. We systematically evaluated the association between clinicopathological features and the expression of the 13 m6A RNA methylation regulators in DTC.

### Prognostic model

We clustered the DTC patients into different groups using R package ConsensusClusterPlus (version 1.49.0, resample rate of 80%, 50 iterations and Pearson correlation, bioconductor.org/packages/devel/bioc/html/ConsensusClusterPlus.html). To discover potential m6A RNA methylation regulators that affect the prognosis of DTC patients, least absolute shrinkage and selection operator (LASSO) Cox regression algorithm 30, 31 was applied using the R package survival (version 2.44; https://CRAN.R-project.org/view=Survival) and R package glmnet (version 2.0-18; https://CRAN.R-project.org/view=Glmnet) to identify optimal prognostic m6A RNA methylation regulators that impact progression-free survival (PFS) of DTC patients. An individual's risk score signature was established as follows:

Risk Score = ∑coefficient (GENEi) × expression (GENEi)

Here, GENEi is the identifier of the ith selected gene. The risk score signature was a measure of prognostic risk for each DTC patient.

### Risk stratification and ROC curve

The risk score was calculated according to the predictive GENE signature model. Using the median risk score as the cutoff, DTC patients were classified into the high-risk group and low-risk group. Kaplan‑Meier analysis was used to generate PFS curves, and log-rank tests were performed to assess PFS differences between high-risk and low-risk groups. The prediction efficiency of the risk signature model was evaluated by calculating the area under the curve (AUC) of the receiver operating characteristic (ROC) curve using R package survival ROC (version 1; https://CRAN.R-project.org/view=Survival ROC). Univariate and multivariate analyses with Cox proportional hazards regression for PFS were performed to determine the prognostic value of the risk score and various clinical characteristics. Hazard ratios (HRs) and 95% confidence intervals (CIs) were estimated.

### GEO database verification

The datasets of DTC patients were obtained from the Gene Expression Omnibus (GEO) database (http://www.ncbi.nlm.nih.gov/geo/). Three mRNA datasets of DTC (GSE33630, GSE35570, GSE60542) were selected in the present study and the expression profiles were normalized by log2‐conversion.

### Statistical analysis

Statistical analyses were performed using SPSS v21.0 software (IBM Corp.). The edger function was used to analyze 13 m6A RNA modification regulators in DTC and normal thyroid tissues. Patients were clustered into different groups by consensus expression of m6A RNA methylation regulators. Chi-square tests were used to evaluate the distribution of clinicopathological characteristics between the two risk groups. In addition, the association between risk groups and the prognosis of patients with DTC was evaluated with the Kaplan‑Meier method and log‑rank test. Prognostic performance was estimated by ROC analysis. Univariate and multivariate Cox regression analyses were used to identify factors that were independently related to the prognosis of DTC patients. The Student's t-test were performed to calculate the results of GEO datasets. P<0.05 was considered statistically significant.

## Results

### Differentially expressed m6A RNA methylation regulators in DTC and normal thyroid

**Figure 1:**
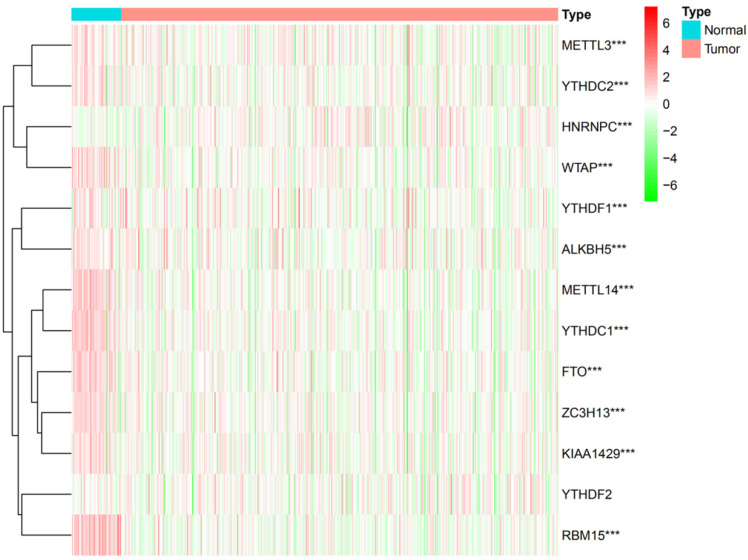
The expression of 13 m6A RNA methylation regulators in DTC and normal thyroid. *P<0.05, **P<0.01, ***P<0.001.

While m6A RNA methylation regulators play a crucial role in the evolution and progression of malignant tumors, little information is available on their expression in DTC. We thus systematically investigated the expressions of 13 m6A RNA methylation regulators in DTC tissue and normal thyroid tissue using gene expression information from TCGA database profiles. The results showed that the expressions of 12 m6A RNA methylation regulators were significantly different between normal thyroid and DTC tissue (P<0.001) (Figure 1).

### Consensus clustering of m6A RNA methylation regulators

**Figure 2:**
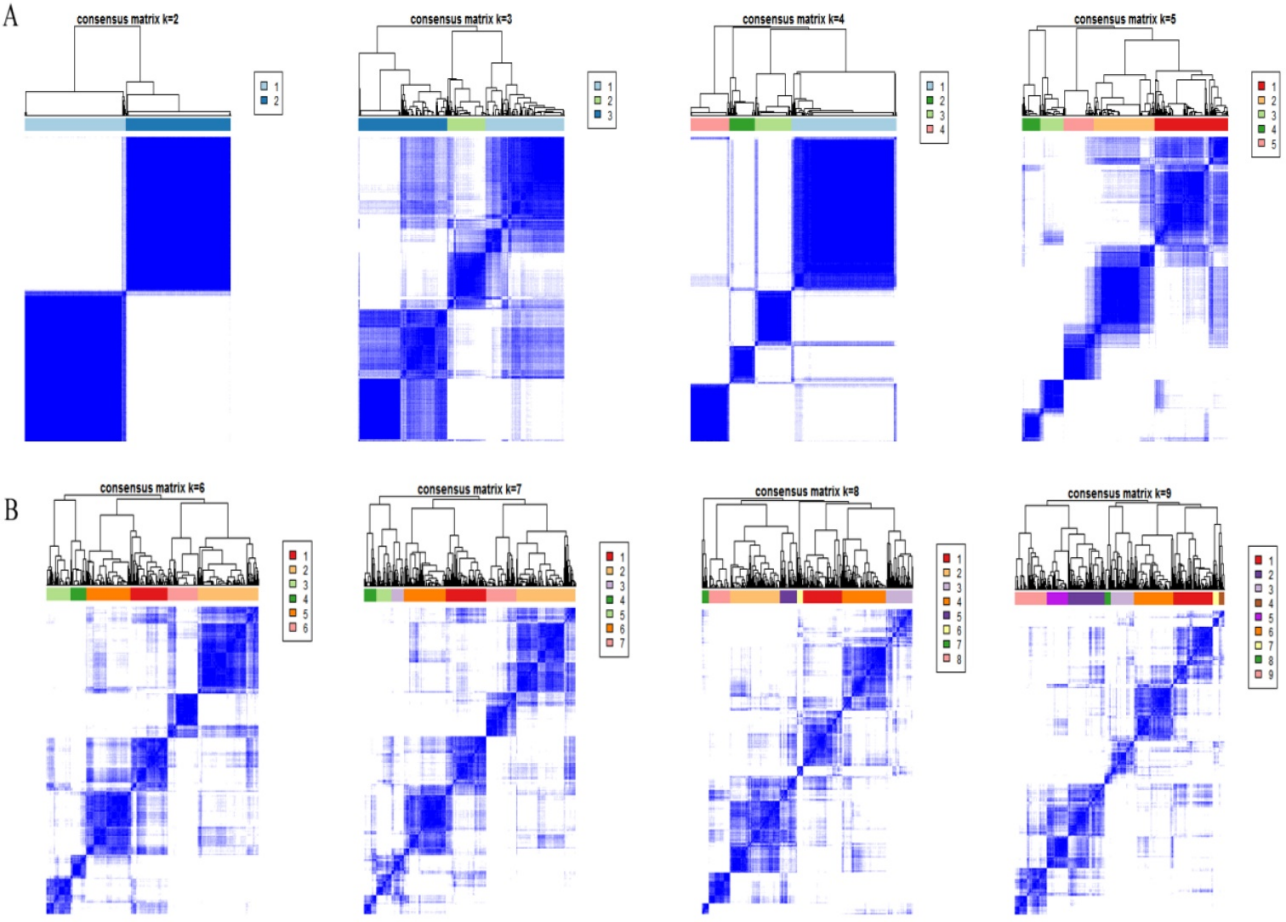
Consensus clustering cumulative distribution feature. (**A**) Consensus clustering cumulative distribution feature for k = 2 to 5. (**B**) Consensus clustering cumulative distribution feature for k = 6 to 9.

**Figure 3:**
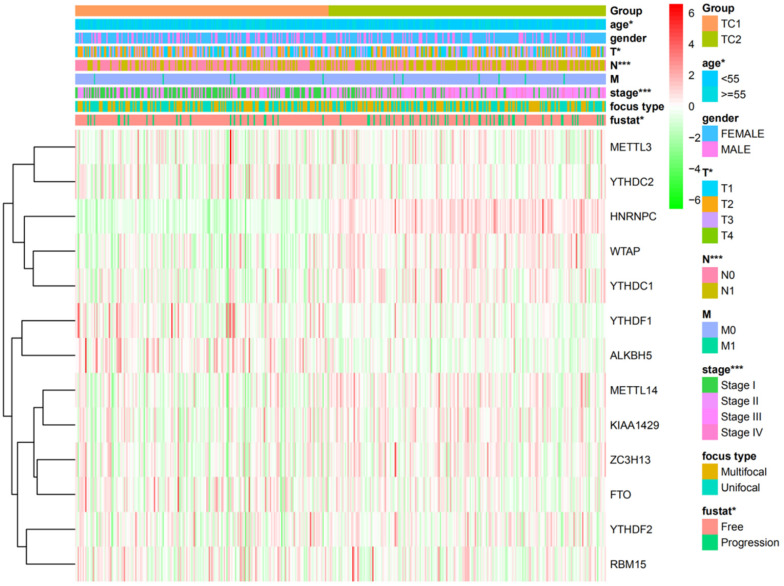
Heatmap and clinicopathologic features of the two clusters (TC1/2).

**Figure 4:**
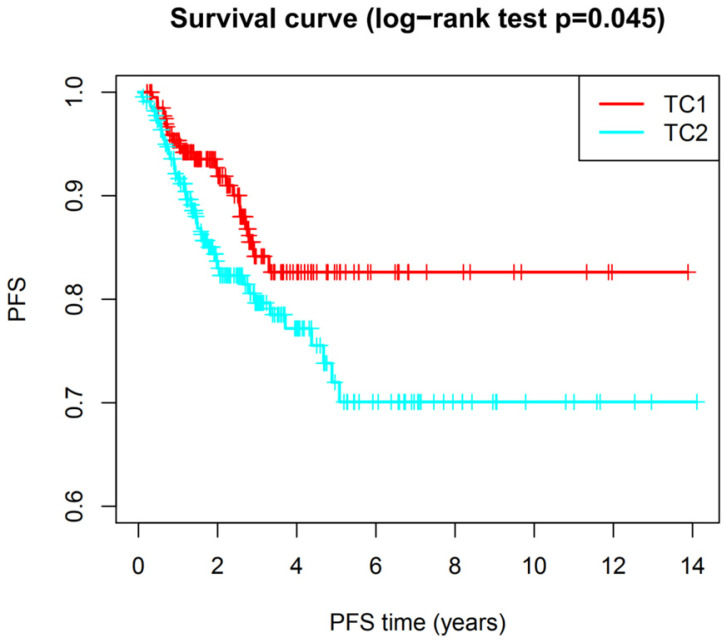
The progression-free survival of DTC between TC1 and TC2. Log-rank test, P<0.05.

Based on the expression similarity of m6A RNA methylation regulators, high intra-group correlation and low inter-group correlation, k=2 was the most appropriate selection with clustering stability increasing from k=2 to 9 in our study (Figure 2A and 2B). Therefore, we divided DTC patients into TC1 or TC2 groups by applying consensus cluster k=2 and analyzed the clinicopathological characteristics between these two subgroups. The TC2 subgroup was significantly associated with an older age at diagnosis (P<0.05), higher T stage (P<0.05), lymph node metastasis (P<0.001), higher TNM stage (P<0.001) and disease-progression state (P<0.05). There were no correlations with other characteristics including gender, focus type, or metastasis stage (P>0.05) (Figure 3). We also investigated the prognosis of DTC patients in TC1 and TC2 subgroups using Kaplan‑Meier and log‑rank test and found that the TC2 subgroup had a shorter PFS compared with the TC1 subgroup (Figure 4; P=0.045).

### Risk model with four selected m6A RNA methylation regulators

**Figure 5:**
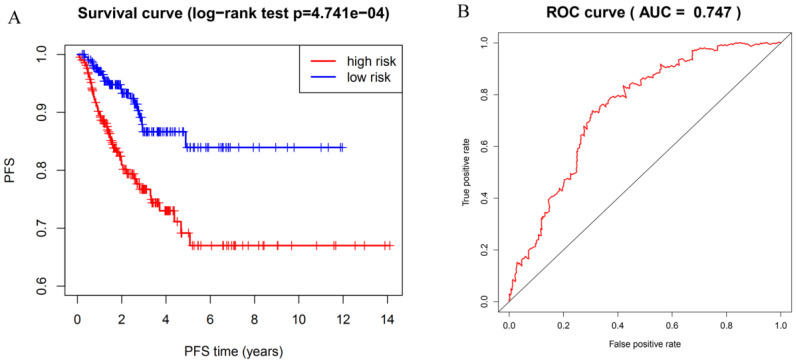
Risk model with four selected m6A RNA methylation regulators. (**A**) Kaplan-Meier curve analysis of progression-free survival in high-risk and low-risk DTC patients. (**B**) Time-dependent ROC curve analysis of the four-gene prognostic risk model.

To identify potential prognostic m6A RNA methylation regulators, we examined the expression data of the twelve difference-expressed genes using the LASSO Cox regression algorithm. According to the minimum criteria, we selected four genes to build the risk signature. The signature was developed as a linear combination of the expression levels of the four genes weighted by their relative regression coefficients in the LASSO algorithm as follows: RS = (0.0341 × expression value of HNRNPC) + (-0.0184 × expression value of ZC3H13) + (-0.0128 × expression value of ALKBH5) + (-0.0877 × expression value of WTAP). We calculated the risk score for each DTC patient based on the risk score model and separated them into high-risk (n = 212) and low-risk groups (n = 213) using the median risk score as the cutoff point. The PFS of the two groups was significantly different; patients in the high-risk group had a shorter PFS compared with patients in low-risk group (Figure 5A; P=4.741e-04). The ROC curve was used to measure the predictive performance of the four-gene prognostic risk model. The AUC of the ROC for the four-gene prognostic model was 0.747 at 5 years of PFS (Figure 5B). These results indicate that the four-gene risk model can accurately predict the outcome of DTC patients.

### Association between prognostic risk scores and clinicopathological features of DTC

**Figure 6:**
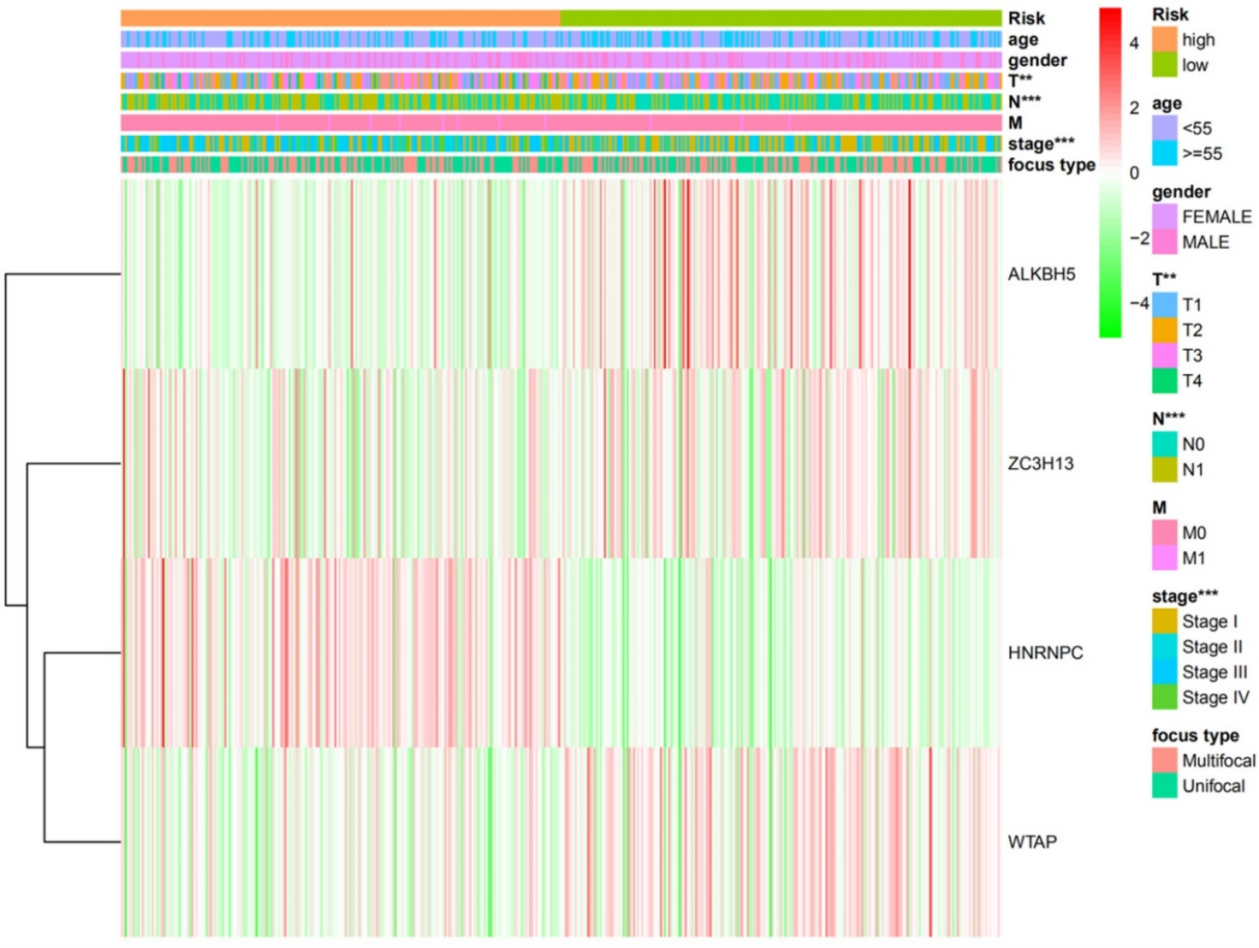
The clinicopathological features and the expression levels of the four m6A RNA methylation regulators in low- and high-risk groups. *P<0.05, **P<0.01, ***P<0.001.

Next, χ^2^ and Fisher exact tests were performed to evaluate whether the prognostic risk scores were connected with clinicopathological features in DTC patients. We found significant differences between the high- and low-risk groups in relation to N grade (P<0.001), T grade (P<0.01), and TNM stage (P<0.001). However, there was no association with other clinicopathological features including gender, age, focus type or M grade (P>0.05). Furthermore, the expression of the four selected m6A RNA methylation regulators in high- and low-risk groups was shown in our study (Figure 6).

### The four-gene risk signature is an independent prognostic indicator

**Figure 7:**
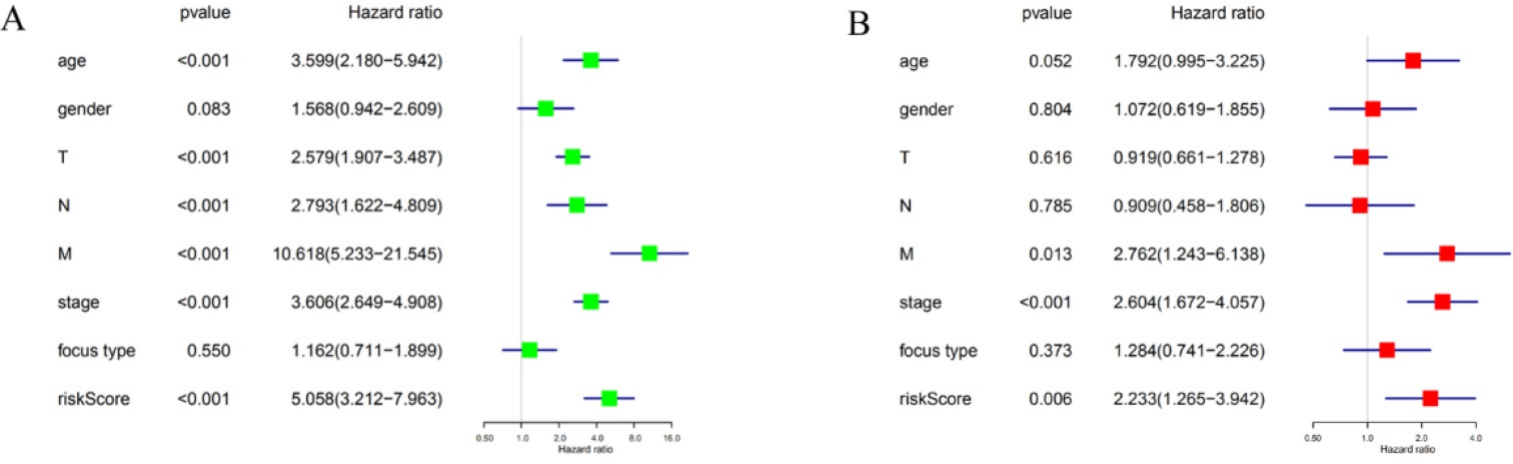
The association between clinicopathological factors and PFS of DTC patients. (**A**) Univariate Cox regression analyses of the association between clinicopathological factors and PFS of DTC patients. (**B**) Multivariate Cox regression analyses of the association between clinicopathological factors and PFS of DTC patients.

**Figure 8:**
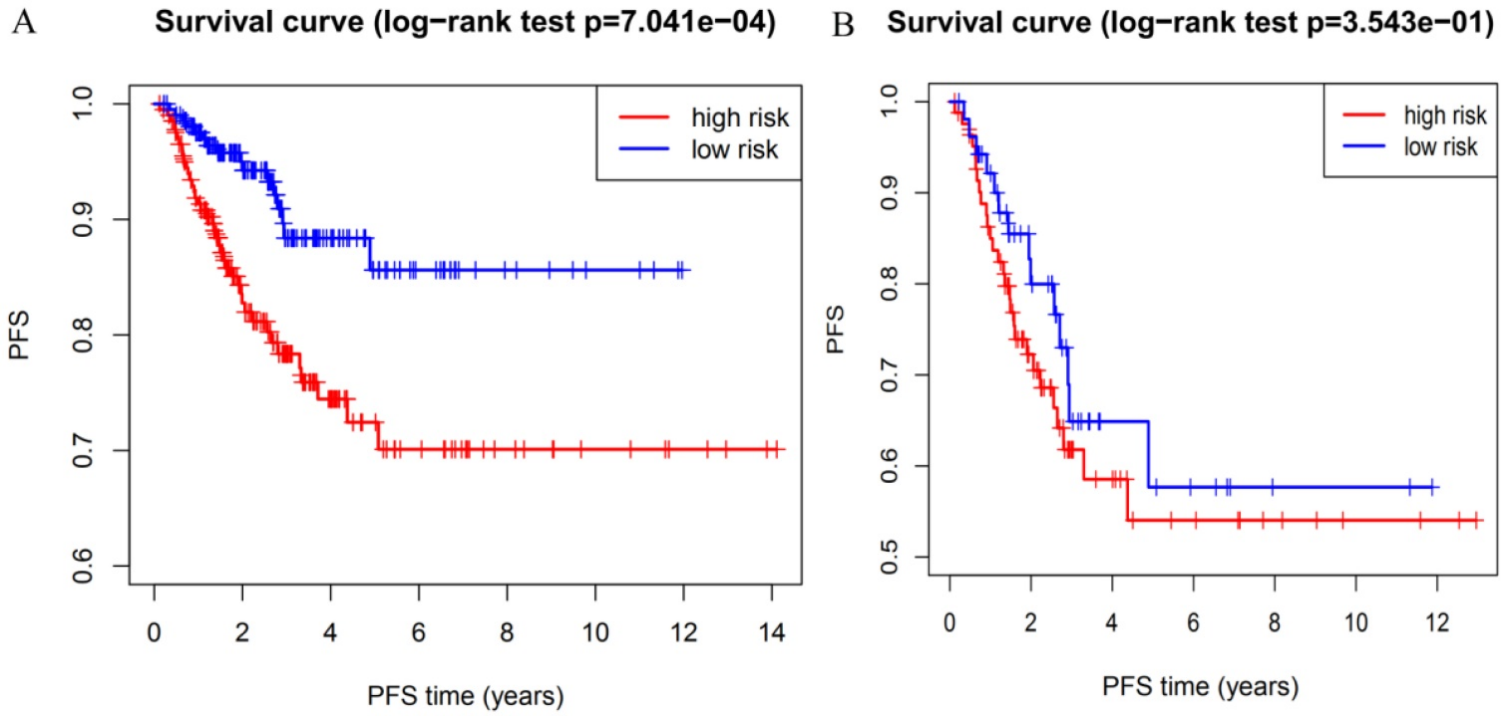
Prognostic value of the risk signature in patients with M grade 0 and TNM grade III. (**A**) Kaplan-Meier PFS curves for patients with M grade 0. (**B**) Kaplan-Meier PFS curves for patients with TNM grade III.

To determine whether the risk signature can independently predict the outcomes of DTC patients, univariate and multivariate Cox analyses were performed. Univariate analysis showed that age, T grade, N grade, M grade, TNM stage and the risk score were all related to PFS (Figure 7A). Including these factors into the multivariate analysis showed that the M grade, TNM stage and the risk score were significantly associated with PFS (Figure 7B). Moreover, DTC patients with high-risk scores had a shorter PFS than those with low risk scores in M grade 0 group (Figure 8A) and TNM stage III groups (Figure 8B). These results confirmed that the four-gene risk signature can predict prognosis of DTC patients independently.

### GEO verification

**Figure 9:**
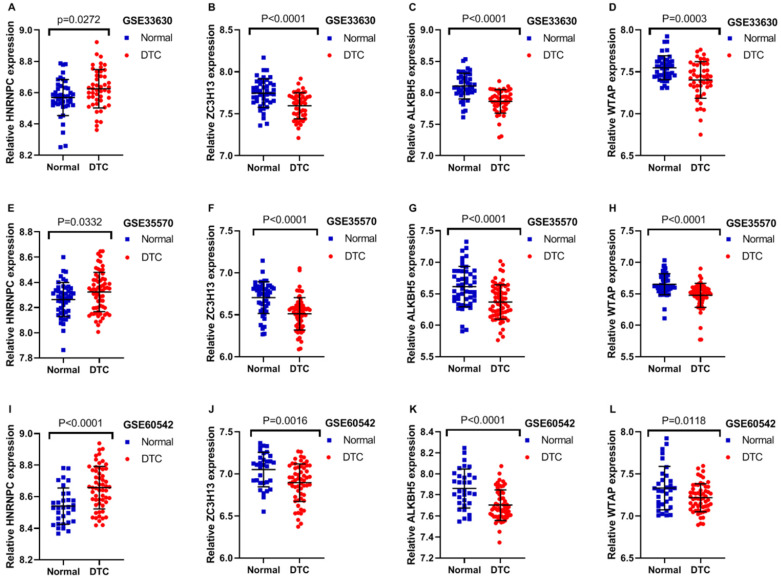
Expression of m6A RNA regulators in different GEO datasets. (**A-D**) The expression levels of four m6A RNA regulators in normal and DTC tissues from the GSE33630. (**E-H**) The expression levels of four m6A RNA regulators in normal and DTC tissues from the GSE35570. (**I-L**) The expression levels of four m6A RNA regulators in normal and DTC tissues from the GSE60542.

In order to further confirm the previous findings from TCGA analysis,we selected three datasets from GEO to verify the accuracy of the above results. The expression profile of these three mRNAs is showed in Table 2. The expression of HNRNPC was significantly higher in DTC than that in normal thyroid tissues as shown in GSE33630, GSE35570 and GSE60542. Furthermore, the results from these three GEO datasets also indicated that the expression of ZC3H13, ALKBH5 and WTAP were all evidently lower in DTC, compared with that in normal thyroid tissues (Figure 9A-L; P<0.05). The outcomes of GEO datasets were consistent with results of the abovementioned TCGA profiles. Unfortunately, no survival information of HNRNPC, ZC3H13, ALKBH5 and WTAP in DTC could be obtained from GEO datasets.

## Discussion

Thyroid carcinoma is the most common endocrine malignancy, with an increasing incidence around the world 32, 33. Even though most thyroid carcinomas are DTCs with favorable prognosis, DTCs display heterogeneity in patients 34, 35. Therefore, novel molecular indicators that could effectively predict and monitor the response to treatment and disease progression of DTCs should be identified to help improve patient care. Recent studies have revealed that m6A modification plays an essential role in various biological processes, including the initiation and progression of cancers 36-38. However, little information has been available regarding the role of m6A in DTCs.

In this study, we found that most known m6A RNA methylation regulators were dysregulated in DTCs. Using consensus cluster, we classified DTC patients into two subgroups (TC1 and TC2). The prognosis and clinicopathological features were significantly different between TC1 and TC2 subgroups. We further developed a risk signature with four m6A RNA methylation regulators that categorized DTC patients into high- and low-risk groups with significantly different PFS.

In eukaryotes, m6A is the most abundant and evolutionarily conserved modification in mRNA and plays a crucial role in several aspects of RNA metabolism 39, 40. Increasing numbers of studies have shown that m6A modifications are controlled by dynamic changes of m6A RNA methylation regulators. Furthermore, dysregulated expression of m6A RNA methylation regulators has been associated with several types of cancers 41-43. For instance, the m6A writer protein METTL3 is a reported oncogene for liver cancer 44, bladder cancer 45 and acute myeloid leukemia 46, but a tumor suppressor for breast cancer 38. Downregulation of the m6A eraser protein ALKBH5 was correlated with poor prognosis in pancreatic cancer 47. However, Zhang *et al* 48 reported that ALKBH5 maintains tumorigenicity of glioblastoma stem-like cells. These findings suggested that m6A RNA methylation regulators may likely have different pathological implications in different diseases.

To explore the role of m6A RNA methylation regulators in DTC patients, we comprehensively analyzed the expression of thirteen regulators in our study. All writers (WTAP, METTL3, METTL14, RBM15, ZC3H13, KIAA1429) were downregulated in DTC compared with normal thyroid tissues. The m6A methylation readers YTHDC1, YTHDC2, and YTHDF1 were expressed significantly lower in DTCs, but the expression of HNRNPC was increased. Both FTO and ALKBH5 m6A methylation erasers were decreased in DTCs. Furthermore, we identified four m6A RNA methylation regulators (WTAP, ZC3H13, HNRNPC and ALKBH5) that were related to the prognosis of DTCs and used these to develop a prognostic signature. In addition, we found that the high-risk group was associated with high N grade, high T grade and high TNM grade, which indicated poor outcomes of DTCs. Univariate and multivariate Cox regression analyses suggested that the four-gene risk signature was an independent prognostic factor for PFS in DTCs. We also found that DTCs with M grade 0 were categorized into high-risk group with shorter PFS or low-risk groups with longer PFS. A similar situation was found in DTCs with TNM stage III, and PFS was significantly different between high- and low-risk groups.

Zinc finger proteins are involved in the regulation of transcription or translation by specific binding of the target molecules. Different combined with DNA, RNA, DNA-RNA, protein, or zinc finger motifs, which result in zinc finger proteins present multifunctional in biological processes 49, 50. ZC3H13 is a classical CCCH zinc finger protein and the encoding gene is located in human chromosome 13q14.13 51. Increasing evidence has shown that ZC3H13 plays an important role in inhibiting tumor progression. In previous research, it has been reported that somatic frame-shift mutation in ZC3H13 gene is detected in colon carcinoma, which suggests that ZC3H13 may be a tumor suppressor 52. Zhu *et al* 53 found that ZC3H13 acts as a tumor suppressor by regulating activation of the Ras-ERK signaling pathway. ALKBH5 is a demethylase that is associated with the regulation of mRNA translation and mRNA metabolism. ALKBH5 is a demethylase that is associated with the regulation of mRNA translation and mRNA metabolism. Increasing evidence indicate that ALKBH5 is implicated in the development of multiple cancers. Overexpression of ALKBH5 has been reported in glioblastoma stem cells and it by stabilizing FOXM1 to promote the proliferation of glioblastoma 48, 54. Zhang C *et al* 55 found that ALKBH5 enhances m6A stability, which increases the levev of m6A in breast carcinoma. Moreover, the number of breast cancer ctem cells can be reduced by ALKBH5 knockdown in breast cancer. He *et al* 47 revealed that ALKBH5 inhibits the motility of pancreatic cancer by downregulating long non-coding RNA KCNK15-AS1 methylation. In our study, we revealed that the expression of ALKBH5 was lower in DTC compared with that in normal thyroid tissues, suggesting that further research is needed to understand the role of ALKBH5 in DTC. WTAP is a nuclear protein widely expressed in cells and tissues, plays an essential role in cellular function and cancer progression, which was first been found for its specific interaction with Wilms' tumor 1 56, 57. WTAP is a conserved nuclear protein that shows decreased expression in breast cancer 38. The reduced expression of WTAP correlated with the accelerated proliferation and invasion of breast cancer. However, a number of recent studies have also shown that WTAP was act as an oncogene and was associated with malignant tumors closely. WTAP negatively regulate WT1.9 to promote tumorigenesis in colorectal cancer 58. HNRNPC is an RNA-binding protein located in the nucleus and the cytoplasm. It is closely related to mRNA metabolism 59, including mRNA splicing, mRNA stabilization and translation.

HNRNPC is aberrantly up-regulated in melanoma 60, glioblastoma 61 and breast cancer 62. In the present study, we demonstrated that the expression of HNRNPC was increased and that of m6A methylases (ZC3H13, ALKBH5 and WTAP) were significantly decreased in DTC tissues compared with normal thyroid tissue. Additionally, we developed a risk signature that divided DTC patients into high-risk and low-risk groups with significantly different PFS times. These results may allow clinicians to determine individualized treatment for DTC patients with different clinical features.

In conclusion, here we revealed the expression and prognostic value of m6A RNA methylation regulators in DTC. This study also provides crucial evidence supporting further research of the role of RNA m6A methylation in DTC.

## Figures and Tables

**Table 1:** Clinical features of 425 DTC patients

Variables	DTC patients (N=425)	
	n	%
**Gender**		
Male	118	27.76
Female	307	72.24
**Age (year)**		0.689
<55	281	66.12
≥55	144	33.88
**TNM stage**		
Stage I	236	55.53
Stage II	43	10.12
Stage III	99	23.29
Stage IV	47	11.06
**Focus type**		
Unifocal	223	52.47
Multifocal	202	47.53
**Vital Status**		
Free	360	84.71
Recurrence	65	15.29

DTC: Differentiated Thyroid Carcinoma.

**Table 2:** Expression data of three mRNA in GEO datasets

GEO datasets	Year	Country	Platform	Sample	N	Relative expression of mRNA							
						HNRNPC		ZC3H13		ALKBH5		WTAP	
						X±S	P	X±S	P	X±S	P	X±S	P
GSE33630	2012	Belgium	GPL570	Normal	44	8.568±0.1152	0.0272	7.746±0.1695	<0.0001	8.108±0.2046	<0.0001	7.549±0.1431	0.0003
				DTC	47	8.625±0.1229		7.596±0.1549		7.863±0.1880		7.403±0.2190	
GSE35570	2015	Poland	GPL570	Normal	48	8.263±0.1357	0.0332	6.705±0.1896	<0.0001	6.615±0.3214	<0.0001	6.653±0.1673	<0.0001
				DTC	65	8.324±0.1558		6.513±0.1935		6.370±0.2733		6.477±0.1909	
GSE60542	2015	Belgium	GPL570	Normal	32	8.540±0.1142	<0.0001	7.051±0.2058	0.0016	7.861±0.1845	<0.0001	7.331±0.2567	0.0118
				DTC	57	8.657±0.1343		6.894±0.2241		7.702±0.1441		7.216±0.1650	

GEO: Gene Expression Omnibus; DTC: Differentiated Thyroid Carcinoma.
